# Bromido(2,2′:6′,2′′-terpyridine)platinum(II) dibromidoaurate(I) dimethyl sulfoxide solvate

**DOI:** 10.1107/S1600536809033248

**Published:** 2009-08-26

**Authors:** Michael I. Kahn, James A. Golen, Arnold L. Rheingold, Linda H. Doerrer

**Affiliations:** a Chemistry Department, Boston University, 590 Commonwealth Ave., Boston, Massachusetts 02215, USA; b Department of Chemistry and Biochemistry, University of Massachusetts–Dartmouth, North Dartmouth, Massachusetts 02747, USA; c Department of Chemistry and Biochemistry, University of California–San Diego, 9500 Gilman Drive, MC 0358, La Jolla, California 92093, USA

## Abstract

The crystal structure of the title compound, [PtBr(C_15_H_11_N_3_)][AuBr_2_]·(CH_3_)_2_SO, exhibits infinite chains of {PtAuPt}_∞_ metallophilic inter­actions along the *b* axis. Two cations and one anion stack in a trimer with a unique Pt⋯Au distance of 3.3361 (5) Å and Pt⋯Pt contacts of 3.4335 (6) Å. The remaining [AuBr_2_]^−^ anion forms no close contacts.

## Related literature

For the related chloride structure, [Pt(tpy)Cl][AuCl_2_] (tpy=2,2′:6′,2"-terpyridine), see Hayoun *et al.* (2006 ▶). For the related [Pt(tpy)I][AuI_2_] complex, see Angle *et al.* (2007 ▶). For a review of double salts with metallophilic inter­actions, see Doerrer (2008 ▶). The synthesis of [Pt(tpy)*X*]*X* complexes (*X* = Cl, Br, I) is discussed in Annibale *et al.* (2004 ▶), and the preparation of [Au*X*_2_]^−^ in Braunstein & Clark (1973 ▶). For background to metallophilic inter­actions, see: Pyykkö (1997 ▶). For a description of the Cambridge Structural Database, see: Allen (2002 ▶).
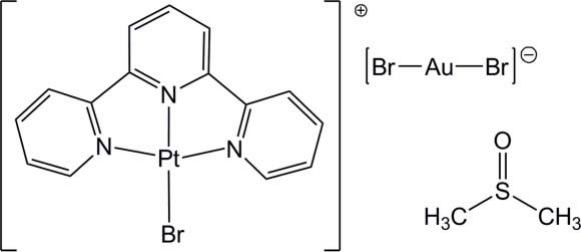


## Experimental

### 

#### Crystal data

[PtBr(C_15_H_11_N_3_)][AuBr_2_]·C_2_H_6_OS
*M* *_r_* = 943.18
Triclinic, 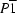

*a* = 8.1463 (11) Å
*b* = 10.0930 (14) Å
*c* = 13.9624 (19) Å
α = 81.905 (2)°
β = 87.675 (2)°
γ = 68.532 (3)°
*V* = 1057.6 (3) Å^3^
*Z* = 2
Mo *K*α radiation
μ = 19.31 mm^−1^
*T* = 208 K
0.30 × 0.20 × 0.15 mm

#### Data collection

Bruker SMART CCD area-detector diffractometer
Absorption correction: multi-scan (*SADABS*; Sheldrick, 2000 ▶) *T*_min_ = 0.068, *T*_max_ = 0.160
7511 measured reflections
4826 independent reflections
4312 reflections with *I* > 2σ(*I*)
*R*_int_ = 0.019

#### Refinement

*R*[*F* ^2^ > 2σ(*F* ^2^)] = 0.039
*wR*(*F* ^2^) = 0.103
*S* = 1.01
4826 reflections
249 parameters
H-atom parameters constrained
Δρ_max_ = 2.01 e Å^−3^
Δρ_min_ = −4.14 e Å^−3^

### 

Data collection: *SMART* (Bruker, 2005 ▶); cell refinement: *SAINT* (Bruker, 2005 ▶); data reduction: *SAINT*; program(s) used to solve structure: *SHELXS97* (Sheldrick, 2008 ▶); program(s) used to refine structure: *SHELXL97* (Sheldrick, 2008 ▶); molecular graphics: *ORTEP-3* (Farrugia, 1997 ▶); software used to prepare material for publication: *SHELXTL* (Sheldrick, 2008 ▶).

## Supplementary Material

Crystal structure: contains datablocks I, global. DOI: 10.1107/S1600536809033248/cv2602sup1.cif

Structure factors: contains datablocks I. DOI: 10.1107/S1600536809033248/cv2602Isup2.hkl

Additional supplementary materials:  crystallographic information; 3D view; checkCIF report

## Figures and Tables

**Table 1:** Selected geometric parameters (Å, °) in [Pt(tpy)*X*][Au*X*_2_], *X* = Cl, Br, I

	Cl	Br	I		
Au—Pt	3.2684 (1)	3.3361 (5)	4.2546 (4)		
Pt—*X*	2.305 (3)	2.4319 (8)	2.5930 (5)		
Au—*X*	2.271 (3)	2.3984 (9)	2.5581 (5)		
Pt—Pt	3.4535 (7)	3.4335 (6)	3.5278 (3)		
					
	Cl	Br			
*X*2—Au1—Pt1	88.63 (7)	81.70 (2)			
	91.37 (7)	98.30 (2)			
*X*1—Pt1—Au1	97.62 (7)	84.08 (2)			
Au1—Pt1—Pt1(1 − *x*, 2 − *y*, −*z*)	165.10 (2)	173.94 (1)			

**Fig. 1.**
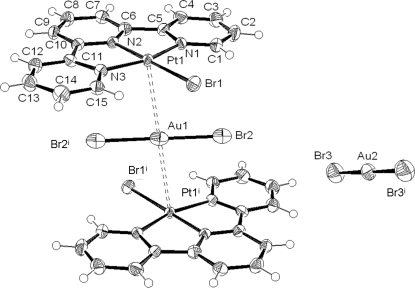
A view of the structure and stacking of two [Pt(tpy)Br]+ cations and one [AuBr2]- anion into a single cation with the second [AuBr2]- anion showing the atomic numbering [symmetry code: (i) -x, 1 - y, -z]. Metallophilic contacts are indicated with dotted lines. Displacement ellipsoids are drawn at the 50% probability level for non-H atoms and one molecule of DMSO has been omitted for clarity.

**Fig. 2.**
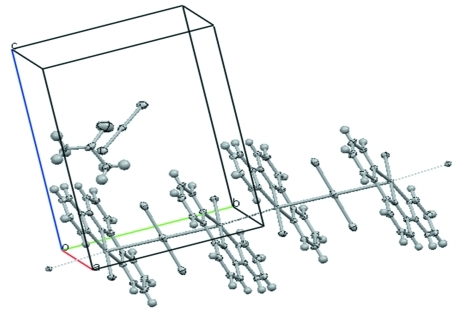
A view of the stacking and structure in (I). Close contacts between {Pt2Au}+ units are shown as dotted lines. Displacement ellipsoids are drawn at the 50% probability level.

## supplementary crystallographic information

### Comment

The title compound, (I), is the bromide analog of the previously published chloride (Hayoun *et al.*, 2006) and iodide (Angle *et al.*, 2007) derivatives.

There are no previous structural characterizations of [Pt(tpy)Br]^+^ (tpy=2,2':6',2"-terpyridine), but the interatomic distances within the [Pt(tpy)]^2+^ are unexceptional and unperturbed by the intermolecular interactions. According to the Cambridge Structural Database (Version 5.30, May 2009; Allen, 2002), the linear [AuBr_2_]^-^ anion has been structurally characterized 32 times with an average Au—Br distance of 2.376 (3) Å and Br—Au—Br angle of 179.3 (2)°, with which the anions in (I) compare favorably. The structure of (I) is analogous to that of [Pt(tpy)Cl]^+^[AuCl_2_]^-^, with metallophilic interactions forming among two platinum(II) and one gold(I) centers to form {[Pt(tpy)Br]_2_[AuBr_2_]}^+^ cations (Figure 1). These cations also form metallophilic interactions among each other resulting in an infinite chain of {PtAuPt}_∞_ metallophilic interactions along the *b*-axis with the remaining [AuBr_2_]^-^ counteranion found outside of the chain (Figure 2). A solvent DMSO molecule was also found in the lattice. The bromide ligands are small enough to allow for metallophilic interactions between gold(I) and platinum(II) centers (Figure 1). No extended metallophilic chains exist in the iodide derivative, which exhibits only pairwise contacts between the cations and between the anions.

As seen in Table 1, the Pt(II)···Pt(II) distances in the bromide derivative are the shortest of all three halogenated species, at 3.4335 (6) Å. The chloride and iodide derivatives exhibit 3.4535 (7) and 3.5278 (3) Å Pt···Pt metallophilic distances, respectively. Evidently the bromide ligand promotes shorter Pt···Pt bonds than the chloride or iodide derivatives, consistent with expectations that more electron rich metal centers promote metallophilic interactions (Pyykkö, 1997). As bromide is softer and less electronegative than chloride, its adjacent platinum center is less electron deficient and bromide is small enough to allow stacking for metallophilic bonding. The gold-platinum distances increase slightly with halogen size from Cl to Br. The Au···Pt···Pt angle is more linear in the bromide derivative at 173.9°, compared to the chloride derivative with an angle of 165.1°. These distances and angles, along with other potentially interesting geometrical values, are collected in Table 1.

The structure of compound (I), therefore, completes a study of the [Pt(tpy)*X*][Au*X*_2_] systems and demonstrates that the halide constituent in the [Pt(tpy)*X*]^+^ ion has a determining effect on the length of the Pt···Pt metallophilic contacts for steric and electronic reasons.

### Experimental

[Pt(tpy)Br]Br, prepared according to the literature (Annibale *et al.*, 2004), was mixed with potassium tetrabromoaurate, KAuBr_4_. Dry acetone was added to the mixture to reduce the gold(III) in KAuBr_4_ to gold(I) in [AuBr_2_]^-^, as expected from the literature (Braunstein and Clark, 1973). This resulted in a maroon solution which turned light orange after stirring for three minutes at 30°C. The solution was allowed to mix at 30°C for four h, resulting in KBr, bromoacetone, and the orange powder [Pt(tpy)Br]^+^ [AuBr_2_]^-^ (in 66% yield) as the products. The orange powder [Pt(tpy)Br]^+^ [AuBr_2_]^-^ was dissolved in DMSO and layered with chloroform to form red block-like crystals.

### Refinement

The crystal was mounted on a CryoLoop with Paratone-N oil and immediately placed under a stream of N~2~ on a Bruker *SMART APEX* CCD system. All H atoms were positioned geometrically (C—H = 0.94–0.97 Å), and allowed to ride on their parent atoms, with Uĩso~ = 1.2–1.5 U~eq~(C). The highest residual peak [2.01 e Å^-3^] and deepest hole [-4.13 e Å^-3^] are situated 0.11 and 0.87 Å from Pt1, respectively.

### Crystal data

**Table:**

[PtBr(C_15_H_11_N_3_)][AuBr_2_]·C_2_H_6_OS	*Z* = 2
*M**_r_* = 943.18	*F*(000) = 852
Triclinic, *P*1	*D*_x_ = 2.962 Mg m^−^^3^
Hall symbol: -P 1	Mo *K*α radiation, λ = 0.71073 Å
*a* = 8.1463 (11) Å	Cell parameters from 5521 reflections
*b* = 10.0930 (14) Å	θ = 2.5–28.2°
*c* = 13.9624 (19) Å	µ = 19.31 mm^−^^1^
α = 81.905 (2)°	*T* = 208 K
β = 87.675 (2)°	Block, red
γ = 68.532 (3)°	0.30 × 0.20 × 0.15 mm
*V* = 1057.6 (3) Å^3^	

### Data collection

**Table:**

Bruker SMART CCD area-detector diffractometer	4826 independent reflections
Radiation source: fine-focus sealed tube	4312 reflections with *I* > 2σ(*I*)
graphite	*R*_int_ = 0.019
φ and ω scans	θ_max_ = 28.2°, θ_min_ = 1.5°
Absorption correction: multi-scan (*SADABS*; Sheldrick, 2000)	*h* = −8→10
*T*_min_ = 0.068, *T*_max_ = 0.160	*k* = −13→10
7511 measured reflections	*l* = −18→17

### Refinement

**Table:**

Refinement on *F*^2^	Primary atom site location: structure-invariant direct methods
Least-squares matrix: full	Secondary atom site location: difference Fourier map
*R*[*F*^2^ > 2σ(*F*^2^)] = 0.039	Hydrogen site location: inferred from neighbouring sites
*wR*(*F*^2^) = 0.103	H-atom parameters constrained
*S* = 1.01	*w* = 1/[σ^2^(*F*_o_^2^) + (0.0756*P*)^2^] *P* = (*F*_o_^2^ + 2*F*_c_^2^)/3
4826 reflections	(Δ/σ)_max_ = 0.001
249 parameters	Δρ_max_ = 2.01 e Å^−^^3^
0 restraints	Δρ_min_ = −4.13 e Å^−^^3^

### Special details

**Table:**

Geometry. All e.s.d.'s (except the e.s.d. in the dihedral angle between two l.s. planes) are estimated using the full covariance matrix. The cell e.s.d.'s are taken into account individually in the estimation of e.s.d.'s in distances, angles and torsion angles; correlations between e.s.d.'s in cell parameters are only used when they are defined by crystal symmetry. An approximate (isotropic) treatment of cell e.s.d.'s is used for estimating e.s.d.'s involving l.s. planes.
Refinement. Refinement of *F*^2^ against ALL reflections. The weighted *R*-factor *wR* and goodness of fit *S* are based on *F*^2^, conventional *R*-factors *R* are based on *F*, with *F* set to zero for negative *F*^2^. The threshold expression of *F*^2^ > σ(*F*^2^) is used only for calculating *R*-factors(gt) *etc*. and is not relevant to the choice of reflections for refinement. *R*-factors based on *F*^2^ are statistically about twice as large as those based on *F*, and *R*- factors based on ALL data will be even larger.

### Fractional atomic coordinates and isotropic or equivalent isotropic displacement parameters (Å^2^)

**Table:**

	*x*	*y*	*z*	*U*_iso_*/*U*_eq_	
Au1	1.0000	0.5000	1.0000	0.03631 (12)	
Au2	0.5000	0.5000	0.5000	0.03754 (12)	
Pt1	0.01155 (3)	0.82805 (2)	−0.005856 (15)	0.02023 (9)	
Br1	−0.25578 (8)	0.87221 (7)	0.08893 (5)	0.03042 (15)	
Br2	0.98737 (9)	0.44194 (7)	1.17203 (6)	0.03918 (18)	
Br3	0.75978 (12)	0.32173 (10)	0.45033 (6)	0.0514 (2)	
S1	0.3410 (3)	1.1786 (2)	0.55776 (14)	0.0477 (5)	
N1	0.1935 (6)	0.7291 (5)	0.1004 (4)	0.0220 (10)	
N3	−0.0990 (6)	0.9177 (5)	−0.1372 (4)	0.0212 (9)	
N2	0.2244 (6)	0.7937 (5)	−0.0823 (4)	0.0196 (9)	
O1	0.3110 (10)	1.0395 (7)	0.5756 (6)	0.079 (2)	
C1	0.1657 (9)	0.6989 (7)	0.1947 (5)	0.0292 (13)	
H1A	0.0493	0.7257	0.2172	0.035*	
C2	0.3038 (10)	0.6292 (8)	0.2598 (5)	0.0363 (15)	
H2A	0.2809	0.6086	0.3256	0.044*	
C3	0.4751 (10)	0.5900 (8)	0.2277 (5)	0.0356 (15)	
H3A	0.5700	0.5420	0.2713	0.043*	
C4	0.5057 (9)	0.6221 (7)	0.1305 (5)	0.0313 (14)	
H4A	0.6216	0.5976	0.1072	0.038*	
C5	0.3634 (8)	0.6908 (6)	0.0682 (4)	0.0239 (12)	
C6	0.3808 (8)	0.7276 (7)	−0.0365 (5)	0.0275 (13)	
C7	0.5327 (8)	0.7028 (6)	−0.0895 (5)	0.0274 (13)	
H7A	0.6440	0.6568	−0.0592	0.033*	
C8	0.5184 (8)	0.7467 (7)	−0.1880 (5)	0.0307 (14)	
H8A	0.6215	0.7295	−0.2249	0.037*	
C9	0.3560 (8)	0.8153 (7)	−0.2337 (4)	0.0270 (12)	
H9A	0.3475	0.8453	−0.3008	0.032*	
C10	0.2060 (8)	0.8385 (6)	−0.1780 (4)	0.0237 (12)	
C11	0.0209 (8)	0.9105 (6)	−0.2094 (4)	0.0241 (12)	
C12	−0.0334 (9)	0.9673 (7)	−0.3032 (5)	0.0306 (14)	
H12A	0.0508	0.9637	−0.3519	0.037*	
C13	−0.2089 (9)	1.0288 (7)	−0.3261 (5)	0.0344 (15)	
H13A	−0.2470	1.0662	−0.3903	0.041*	
C14	−0.3288 (9)	1.0348 (8)	−0.2530 (5)	0.0356 (15)	
H14A	−0.4502	1.0775	−0.2672	0.043*	
C15	−0.2725 (8)	0.9788 (6)	−0.1592 (5)	0.0270 (13)	
H15A	−0.3559	0.9833	−0.1100	0.032*	
C16	0.2828 (11)	1.2500 (9)	0.4364 (5)	0.0473 (19)	
H16A	0.3572	1.1838	0.3945	0.071*	
H16B	0.2987	1.3414	0.4222	0.071*	
H16C	0.1603	1.2642	0.4255	0.071*	
C17	0.1620 (13)	1.3074 (11)	0.6114 (7)	0.063 (3)	
H17A	0.1799	1.2924	0.6809	0.094*	
H17B	0.0525	1.2965	0.5969	0.094*	
H17C	0.1560	1.4035	0.5856	0.094*	

### Atomic displacement parameters (Å^2^)

**Table:**

	*U*^11^	*U*^22^	*U*^33^	*U*^12^	*U*^13^	*U*^23^
Au1	0.02295 (18)	0.0324 (2)	0.0533 (3)	−0.00747 (15)	−0.00103 (16)	−0.01197 (17)
Au2	0.0434 (2)	0.0484 (2)	0.0243 (2)	−0.02176 (18)	−0.00643 (16)	0.00001 (16)
Pt1	0.01865 (13)	0.02448 (13)	0.01805 (14)	−0.00871 (9)	0.00074 (9)	−0.00222 (9)
Br1	0.0254 (3)	0.0345 (3)	0.0311 (3)	−0.0109 (3)	0.0069 (2)	−0.0054 (3)
Br2	0.0306 (3)	0.0323 (3)	0.0542 (5)	−0.0095 (3)	−0.0011 (3)	−0.0097 (3)
Br3	0.0499 (5)	0.0579 (5)	0.0436 (5)	−0.0160 (4)	−0.0025 (4)	−0.0072 (4)
S1	0.0383 (9)	0.0678 (13)	0.0308 (10)	−0.0188 (9)	−0.0043 (8)	0.0137 (9)
N1	0.021 (2)	0.027 (2)	0.019 (2)	−0.0096 (19)	−0.0004 (19)	−0.0027 (19)
N3	0.017 (2)	0.022 (2)	0.022 (2)	−0.0041 (18)	−0.0033 (18)	−0.0013 (19)
N2	0.018 (2)	0.021 (2)	0.020 (2)	−0.0077 (18)	0.0020 (18)	−0.0030 (19)
O1	0.081 (5)	0.057 (4)	0.079 (5)	−0.020 (4)	0.013 (4)	0.033 (4)
C1	0.034 (3)	0.035 (3)	0.021 (3)	−0.017 (3)	0.003 (3)	−0.002 (3)
C2	0.042 (4)	0.045 (4)	0.023 (3)	−0.020 (3)	−0.001 (3)	0.005 (3)
C3	0.039 (4)	0.038 (4)	0.029 (4)	−0.015 (3)	−0.012 (3)	0.006 (3)
C4	0.030 (3)	0.034 (3)	0.032 (4)	−0.013 (3)	−0.004 (3)	−0.004 (3)
C5	0.023 (3)	0.025 (3)	0.024 (3)	−0.011 (2)	0.000 (2)	−0.001 (2)
C6	0.024 (3)	0.030 (3)	0.028 (3)	−0.010 (2)	−0.001 (2)	−0.003 (3)
C7	0.023 (3)	0.029 (3)	0.029 (3)	−0.008 (2)	−0.002 (2)	−0.004 (3)
C8	0.024 (3)	0.036 (3)	0.032 (4)	−0.011 (3)	0.007 (3)	−0.005 (3)
C9	0.027 (3)	0.035 (3)	0.020 (3)	−0.013 (3)	0.002 (2)	−0.002 (2)
C10	0.025 (3)	0.023 (3)	0.025 (3)	−0.010 (2)	0.001 (2)	−0.003 (2)
C11	0.022 (3)	0.026 (3)	0.025 (3)	−0.008 (2)	−0.001 (2)	−0.007 (2)
C12	0.036 (3)	0.029 (3)	0.026 (3)	−0.013 (3)	−0.003 (3)	0.001 (3)
C13	0.035 (3)	0.036 (3)	0.029 (4)	−0.010 (3)	−0.009 (3)	0.001 (3)
C14	0.027 (3)	0.042 (4)	0.035 (4)	−0.010 (3)	−0.010 (3)	0.002 (3)
C15	0.022 (3)	0.030 (3)	0.029 (3)	−0.009 (2)	−0.003 (2)	−0.003 (3)
C16	0.057 (5)	0.060 (5)	0.028 (4)	−0.025 (4)	0.001 (3)	−0.001 (3)
C17	0.070 (6)	0.084 (7)	0.049 (5)	−0.040 (5)	0.016 (5)	−0.025 (5)

### Geometric parameters (Å, °)

**Table:**

Au1—Br2^i^	2.3981 (9)	C4—H4A	0.9400
Au1—Br2	2.3981 (9)	C5—C6	1.471 (9)
Au2—Br3	2.3753 (9)	C6—C7	1.375 (9)
Au2—Br3^ii^	2.3753 (9)	C7—C8	1.381 (9)
Pt1—N2	1.944 (5)	C7—H7A	0.9400
Pt1—N3	2.015 (5)	C8—C9	1.383 (9)
Pt1—N1	2.018 (5)	C8—H8A	0.9400
Pt1—Br1	2.4320 (7)	C9—C10	1.385 (8)
S1—O1	1.497 (7)	C9—H9A	0.9400
S1—C16	1.757 (8)	C10—C11	1.468 (8)
S1—C17	1.782 (9)	C11—C12	1.376 (9)
N1—C1	1.338 (8)	C12—C13	1.365 (9)
N1—C5	1.369 (7)	C12—H12A	0.9400
N3—C15	1.348 (7)	C13—C14	1.376 (10)
N3—C11	1.366 (8)	C13—H13A	0.9400
N2—C6	1.342 (7)	C14—C15	1.376 (9)
N2—C10	1.345 (8)	C14—H14A	0.9400
C1—C2	1.382 (10)	C15—H15A	0.9400
C1—H1A	0.9400	C16—H16A	0.9700
C2—C3	1.379 (10)	C16—H16B	0.9700
C2—H2A	0.9400	C16—H16C	0.9700
C3—C4	1.385 (9)	C17—H17A	0.9700
C3—H3A	0.9400	C17—H17B	0.9700
C4—C5	1.380 (9)	C17—H17C	0.9700
			
Br2^i^—Au1—Br2	180.0	C6—C7—H7A	120.7
Br3—Au2—Br3^ii^	180.000 (1)	C8—C7—H7A	120.7
N2—Pt1—N3	80.7 (2)	C7—C8—C9	121.5 (6)
N2—Pt1—N1	80.8 (2)	C7—C8—H8A	119.2
N3—Pt1—N1	161.5 (2)	C9—C8—H8A	119.2
N2—Pt1—Br1	179.63 (16)	C8—C9—C10	118.2 (6)
N3—Pt1—Br1	98.95 (14)	C8—C9—H9A	120.9
N1—Pt1—Br1	99.58 (14)	C10—C9—H9A	120.9
O1—S1—C16	107.0 (4)	N2—C10—C9	118.9 (5)
O1—S1—C17	106.8 (4)	N2—C10—C11	113.0 (5)
C16—S1—C17	97.3 (4)	C9—C10—C11	128.2 (6)
C1—N1—C5	118.9 (5)	N3—C11—C12	120.8 (6)
C1—N1—Pt1	127.8 (4)	N3—C11—C10	114.7 (5)
C5—N1—Pt1	113.3 (4)	C12—C11—C10	124.4 (6)
C15—N3—C11	119.1 (5)	C13—C12—C11	120.3 (7)
C15—N3—Pt1	127.2 (4)	C13—C12—H12A	119.8
C11—N3—Pt1	113.6 (4)	C11—C12—H12A	119.8
C6—N2—C10	123.9 (5)	C12—C13—C14	118.4 (6)
C6—N2—Pt1	118.2 (4)	C12—C13—H13A	120.8
C10—N2—Pt1	117.9 (4)	C14—C13—H13A	120.8
N1—C1—C2	121.7 (6)	C13—C14—C15	120.6 (6)
N1—C1—H1A	119.2	C13—C14—H14A	119.7
C2—C1—H1A	119.2	C15—C14—H14A	119.7
C3—C2—C1	119.7 (6)	N3—C15—C14	120.7 (6)
C3—C2—H2A	120.1	N3—C15—H15A	119.7
C1—C2—H2A	120.1	C14—C15—H15A	119.7
C2—C3—C4	119.2 (6)	S1—C16—H16A	109.5
C2—C3—H3A	120.4	S1—C16—H16B	109.5
C4—C3—H3A	120.4	H16A—C16—H16B	109.5
C5—C4—C3	118.9 (6)	S1—C16—H16C	109.5
C5—C4—H4A	120.5	H16A—C16—H16C	109.5
C3—C4—H4A	120.5	H16B—C16—H16C	109.5
N1—C5—C4	121.6 (6)	S1—C17—H17A	109.5
N1—C5—C6	115.0 (5)	S1—C17—H17B	109.5
C4—C5—C6	123.4 (6)	H17A—C17—H17B	109.5
N2—C6—C7	119.0 (6)	S1—C17—H17C	109.5
N2—C6—C5	112.7 (5)	H17A—C17—H17C	109.5
C7—C6—C5	128.3 (6)	H17B—C17—H17C	109.5
C6—C7—C8	118.6 (6)		

Symmetry codes: (i) −*x*+2, −*y*+1, −*z*+2; (ii) −*x*+1, −*y*+1, −*z*+1.

### Table 1 Selected geometric parameters (Å, °) in [Pt(tpy)X][AuX_2_], X = Cl, Br, I.

**Table:**

	Cl	Br	I		
Au—Pt	3.2684 (1)	3.3361 (5)	4.2546 (4)		
Pt—X	2.305 (3)	2.4319 (8)	2.5930 (5)		
Au—X	2.271 (3)	2.3984 (9)	2.5581 (5)		
Pt—Pt	3.4535 (7)	3.4335 (6)	3.5278 (3)		
					
	Cl	Br			
X2—Au1—Pt1	88.63 (7)	81.70 (2)			
	91.37 (7)	98.30 (2)			
X1—Pt1—Au1	97.62 (7)	84.08 (2)			
Au1—Pt1—Pt1(1-x, 2-y, -z)	165.10 (2)	173.94 (1)			
